# In vitro antibacterial activity and synergetic effect of crude extract of the *Wohlfahrtia nuba* (Diptera: Sarcophagidae) flesh fly larvae

**DOI:** 10.1007/s42770-023-01024-z

**Published:** 2023-06-20

**Authors:** Azza M. Khedre, Tarek G. Ismail, Gehad A. Hashem, Islam M. Zakaria

**Affiliations:** 1 Zoology Department, Faculty of Science, Sohag University, P.O. Box: 82524, Sohag, Egypt; 2 Microbiology Department, Animal Health Research Institute, Sohag, Egypt; 3 Bacteriology Department, Animal Health Research Institute, Agriculture Research Center, Giza, Egypt

**Keywords:** Antibacterial activity, Synergetic effect, Crude extract, Flesh fly, *Wohlfahrtia nuba*

## Abstract

Multidrug-resistant pathogens have become ubiquitous, and effective treatment alternatives are urgently required. Maggot therapy is a promising agent that is being studied to overcome antibiotic-resistant pathogens. This study evaluated the antibacterial activity of the larvae extract of the *Wohlfahrtia nuba* (wiedmann) (Diptera: Sarcophagidae) flesh fly on the growth of five pathogenic bacterial species (methicillin-sensitive *Staphylococcus aureus* [ATCC 29213], methicillin-resistant *Staphylococcus aureus* [ATCC BAA-1680], *Pseudomonas aeruginosa* [ATCC 27853], *Escherichia coli* [ATCC 25922], and *Salmonella typhi* [ATCC 19430]) in vitro by using different techniques*.* Resazurin-based turbidimetric assay demonstrated that the *W. nuba* maggot exosecretion (ES) was potent against all the bacterial species tested, and according to the determined minimum inhibitory concentration (MIC) for each bacterium, gram-negative bacteria were more sensitive than gram-positive bacteria. Additionally, colony-forming unit assay showed that maggot ES was able to inhibit bacterial growth rate for all bacterial species tested, where the highest bacterial reduction was observed with methicillin-sensitive *S. aureus* (MSSA) followed by *S. typhi*. Moreover, maggot ES was shown to be concentration-dependent, where 100 μL of ES at 200 mg/mL was bactericidal towards methicillin-resistant *S. aureus* (MRSA) and *P. aeruginosa* compared with 100 μL at the MIC of the ES. Moreover, based on the result of agar disc diffusion assay, maggot extract was more efficient against *P. aeruginosa* and *E. coli* than the remaining reference strains tested. Furthermore, the combination between regular antibiotics with maggot ES at different concentrations indicated that ES acts synergistically with the tested antibiotics against the five bacterial models.

**Supplementary Information:**

The online version contains supplementary material available at 10.1007/s42770-023-01024-z.

## Introduction

The extensive use of antibiotics in medical treatments and animal feed has contributed to the emergence of multidrug-resistant pathogens [1]. Three major mechanisms may be responsible for bacterial resistance to antibiotics: mutation, physiological adaptation, and transfer of resistance genes [2]. Therefore, effective alternatives are urgently required to be used instead of antibiotics. One of these alternatives is maggot therapy.

Maggot therapy is a conventional and complementary medicine practice with a history of application from ancient times [3]. It is referred to as biosurgery, which involves the application of the sterile maggots of some flies (e.g., *Lucilia sericata*) to infected, non-healing wounds of humans or animals that did not respond to other forms of therapy, to remove chronic tissues, decrease the risk of infection, and improve wound healing [4].

Generally, insects are extensively used in traditional medicine worldwide [5] because they have a unique innate immune system with a specific pattern of antimicrobial peptide (AMP) production as a response to pathogenic infections. AMPs are produced in all life stages of insects, and the highest activity of AMPs was determined in the final instar larva stage [6].

AMPs are short bioactive molecules which have a varying number of amino acids (from five to over a hundred). They have become of considerable importance to the pharmaceutical industry because they possess rapid and wide-spectrum antimicrobial activities against pathogens including bacteria, viruses, fungi, and parasites [7] with an ability to avoid microbial resistance [8]. Therefore, these peptides seem to be an ideal candidate to help in the combat against drug-resistant bacteria [9].

The discovery of the antibacterial activity of maggot extracts dates back to the 1930s by Simmons. After that, several investigations have focused on the antibacterial activity of maggots’ excretion/secretion (ES) of different species of blowflies against a range of both gram-positive and gram-negative bacteria [10–17]. However, few studies were carried out on flesh fly species [18, 19]. Previous researchers reported that maggot ES have antimicrobial components that inhibit the growth of some bacterial species that are becoming resistant to available antibiotics.

One of the most abundant species of flesh flies present in the warm climate of Sohag Governorate, Egypt, is *Wohlfahrtia nuba*. It was demonstrated previously as a good species to use in maggot therapy [20]. No studies were made to investigate the in vitro antibacterial aspects of *W. nuba* maggot ES following records of marked activity against wound bacteria. Therefore, the objectives of this study were to evaluate the in vitro potential of *W. nuba* maggot ES, as an antibacterial agent, and to estimate the effect of the combination between antibiotics at minimum inhibitory concentration levels (MICs) with maggot ES against both gram-positive and gram-negative bacteria.

## Materials and methods

### Rearing laboratory flies

*Wohlfahrtia nuba* was captured from cattle barns in Sohag Governorate, Egypt. Then, the colony of *W. nuba* flies was maintained under laboratory conditions of temperature (27 ± 3 °C), relative humidity (30 ± 5%), and no attempt was made to regulate the photoperiods. Adults had a continual supply of granulated sugar and water in (30 × 30 × 30 cm) wooden cages with three sides of wire. Females were allowed to larviposit on fresh beef meat. Deposited larvae were collected periodically using forceps from the cages, reared on raw beef for about 3 days in an incubator at 30 °C, and disinfected based on a previously published method [9, 21]. Third instar larvae were used for obtaining the extract since they yield the largest amount of crude ES [11].

### Extraction of larval ES

The extraction method we applied was modified from previously published methods [9, 22]. The collected third instar larvae (100 larvae) were washed with 70% ethanol in a clean Petri dish for 3 min and rinsed with sterile deionized water three times successively. Washed larvae were dried with sterile paper towels and transferred into a clean beaker. The larvae were then incubated at 30 °C with 10 mL of deionized water for 1 h, then covered and tied with mesh cloth. Thereafter, the water was removed by pipette, and the larvae were deactivated by placement in a freezer for 5 min. The water (now containing some exosecretions) was added to the deactivated larvae and gently homogenized manually using a porcelain mortar to disrupt the tissues and expose the cellular content and gut secretions, following the suggestion that antibacterial factors exist in the gut [14]. Then, the resulting homogenate is transferred to 50 mL centrifuge tubes and centrifuged at 4000 *g* for 30 min at 4 °C to remove particulate materials [11]. The resultant supernatant was collected into a clean glass beaker. Lastly, the supernatant was lyophilized to withstand long-term storage and to avoid losses of antibacterial activity [9]. Afterward, the lyophilized ES material was stored at −40 °C. Prior to use, the freeze-dried material was dissolved in deionized water at a final concentration of 200 mg/mL and then filtrated through a cellulose acetate membrane syringe filter with a pore size of 0.22 μm.

### Bacterial strains and preparation of bacterial suspensions

The bacterial species were chosen based on their association with body decomposition [16] and their responsibility for many infections [23, 24].

### Bacterial strains

Five pathogenic bacterial strains were used for the antibacterial assays.

The gram-positive bacterial strains were:

Methicillin-sensitive *Staphylococcus aureus* (MSSA) ATCC 29213.

Methicillin-resistant *Staphylococcus aureus* (MRSA) ATCC BAA-1680.

The gram-negative bacterial strains were:

*Pseudomonas aeruginosa* (*P. aeruginosa*) ATCC 27853.

*Escherichia coli* (*E. coli*) ATCC 25922.

*Salmonella typhi* (*S. typhi*) ATCC 19430.

All the tested strains were obtained from the Animal Health Research Institute, El-Dokky, Giza, Egypt. These strains were maintained on nutrient agar (Oxoid, Singapore) and then incubated at 37 °C for 24 h. Three to four discrete bacterial colonies with similar morphology were inoculated into 10 mL sterile Mueller Hinton broth (MHB) and incubated overnight at 37 °C. The overnight bacterial suspensions were adjusted to 1.5 × 10^8^ cell/mL McFarland Standard, then diluted up to 1.5 × 10^6^ cell/mL.

### Antimicrobial agents

Three antibiotics were used in different assays. These included vancomycin for MSSA and MRSA, gentamycin for *E. coli* and *P. aeruginosa*, and ciprofloxacin for *S. typhi.* All three antibiotics were obtained from Oxoid Ltd. (Basingstoke, Hampshire, England).

### Determination of MIC by using resazurin-based turbidimetric assay

Resazurin solution was prepared according to the method used by Teh et al. [22]. To guarantee homogeneity, a vortex mixer was used to mix the solution for 1 h. The preparation processes were undertaken in the dark, and the solution was kept in a black bottle to avoid light exposure. The present assay was performed to determine the antibacterial activity of the maggot ES of *W. nuba* against previously prepared gram-positive and gram-negative bacteria. Broth microdilution was employed according to the Clinical and Laboratory Standards Institute (CLSI) protocol [25]. A sterile 96-well microtiter plate was used and labelled. A volume of 100 μL of MHB was added to each well of a 96-well plate. For each bacterial culture, each vertical column was prepared as follows: C1 contained 100 μL of sterile distilled water (DW); C2 contained 100 μL of sterile maggot ES (200 mg/mL), and C3 contained 100 μL of sterile DW. Both C1 and C2, as well as C3, represented sterility control columns; however, C4 contained 100 μL of bacterial growth control. The fifth column C5 contained 100 μL of antibiotic (100 mg/mL), which was chosen according to the type of bacterium as previously described. The last column C6 contained 100 μL of sterile maggot ES (200 mg/mL). Both C5 and C6 represented test columns. Thorough mixing was performed in the first well and then double fold serial dilution was performed up to the eighth well using a separate and sterile pipette. Finally, 100 μL of the mixture was discarded from the last well. The obtained concentrations were in the range of 100 to 1.56 mg/mL. After that, 5 μL of diluted bacterial suspension was added to all the wells except the sterile columns to achieve a concentration of 0.5 × 10^5^ cell/mL. Microdilution was performed in triplicates for each bacterial species. The microtiter plates were incubated at 37 °C overnight; then 5 μL of resazurin indicator (6.75 mg/mL) was added to all wells and incubated at 37 °C for another 4 h. Any color changes from blue to pink or colorless were recorded as positive. The lowest concentration prior to color change was recorded as the MIC.

### Determination of antibacterial properties of larval extract

In order to detect the antibacterial properties (bactericidal or bacteriostatic) of larval extracts, a loopful of aliquots from the MIC wells was put in brain heart infusion agar (BHIA) and incubated at 37 °C for 24 h. If bacteria failed to exhibit growth on BHIA after an overnight incubation, the larval extract was recorded to be bactericidal; otherwise, it was considered bacteriostatic.

### Quantification of antibacterial activity of ES using MIC

In order to quantify the antibacterial activity of *W. nuba* extract against the five tested bacterial species, liquid culture assay was performed. Firstly, bacterial suspensions were prepared in 0.5 McFarland scale, and from this suspension, final dilutions of 1.5 × 10^6^ cell/mL were obtained using tenfold serial dilutions. *W. nuba* larval extract was prepared according to its MICs against tested bacterial species as determined from resazurin-based turbidimetric assay from the present study. Secondly, gentamycin, vancomycin, and ciprofloxacin were prepared at MICs against tested bacterial species according to an established method [25]. The experiments were divided into three groups: (1) CONT-BAC group containing 1.5 × 10^6^ cell/mL of bacterial suspensions tested, (2) ES + BAC group containing filter sterilized *W. nuba* larval extract and bacterial suspensions, and (3) CONT-ANTI group containing prepared concentrations of the antibiotics and bacterial suspensions. Subsequently, 100 μL of aliquots from each experimental group (CONT-BAC, ES+BAC, and CONT-ANTI) was plated by spreading onto Petri dishes containing BHI agar at 0, 2, 4, 6, 10, and 24 h. Petri dishes were incubated at 37 °C for 24 h according to the reading period set; then the counting CFU/mL was performed manually.

### Quantification of antibacterial activity of ES using different doses at a concentration (200 mg/mL)

Volumes of 100 μL and 300 μL of *W. nuba* ES (200 mg/mL) were added to 3 mL of bacterial species suspensions diluted 10-fold serially to 0.5 × 10^5^ cell/mL. Deionized water was used in the control experiment, and 100 μL of the test and control was applied onto Muller Hinton agar for plate counting at 0 time and after incubation of the test and control at 37 °C for 18 h. Bacterial cell counts were determined by counting the colonies after the plates were incubated at 37 °C for 24 h.

### Disc diffusion assay

The modified Kirby–Bauer method [26] was used to evaluate the susceptibility of the tested bacterial species to the ES extract. All test strains were grown 24 h prior to performing the assay. A sterile cotton wool swab was dipped into a bacterial suspension adjusted to 0.5 McFarland standard and spread evenly over the entire surface of the BHI agar in 25 mL sterile Petri dishes (90 mm in diameter) under aseptic conditions. Then, 20 μL of the tested extract (200 mg/mL concentration) was added to 6.0 mm blank paper discs (Schleicher & Schuell Bio Science GmbH), and discs were allowed to dry for 3 h at room temperature inside laminar flow. Discs were then placed on agar and plates were incubated at 37 °C for 24 h. The radial zones of inhibition in (mm) were measured. For positive controls, blank paper discs were prepared using 20 μL of gentamycin, vancomycin, and ciprofloxacin at MICs according to CLSI [25], and sterile double-distilled water (d. dH_2_O) was used as a negative control. The assay was done in triplicates.

### Evaluation of synergistic effect between the larval extract and antibiotics

Overnight, five bacterial cultures were sub-cultured in BHI broth to a standard inoculum of 0.5 McFarland turbidity. Then 100 μL of each bacterial solution was inoculated on the surface of BHI agar plates. Serial volumes of maggot extract (25, 20, 15, 10, and 5 μL) at a concentration of 200 mg/mL were prepared. The same volumes of tested antibiotics, gentamycin for *E. coli* and *P. aeruginosa*, vancomycin for MSSA and MRSA, and ciprofloxacin for *S. typhi* were prepared at MIC according to CLSI standards [25]. Subsequently, six sterile paper discs (diameter 6 mm) were spotted into the surface of each inoculated plate. In each plate, a total volume (25 μL) of larval extract was added gradually to one of the discs and allowed to dry for 3 h under a complete septic condition inside the laminar cabinet to achieve 100% larval extract concentration. By the same method, 25 μL of the tested antibiotic was added to the second disc to obtain 100% antibiotic concentration. The remaining four discs contained a mixture of larval extract and antibiotic with different volumes. The resultant antibiotic concentration in the discs of each plate was (100%, 80%, 60%, 40%, 20%, and 0%) to evaluate the susceptibility of survival bacteria against a combination of the suitable antibiotic and larval ES that displayed antibacterial activity. Then, the plates were incubated at 37 °C for 24 h. The diameter of clearing inhibition zones was measured. The experiment was repeated three times.

### Statistical analysis

The results are presented as the mean ± standard error. SPSS software (version 22.0) was used for data analysis. Antibacterial activity of ES in the colony-forming unit was evaluated using the chi-square test, while the rest of the data was analyzed using one-way analysis of variance (ANOVA).

## Results

### Resazurin-based turbidimetric assay

The results of resazurin-based turbidimetric assay indicated that all sterility control wells for all bacterial species tested remained a blue color after overnight incubation and followed by 4 h incubation with resazurin. However, all wells in the growth control columns of all the tested bacteria changed from blue to pink (see supplementary Data S1–S3 for microtiter plate of MSSA, MRSA, *E. coli*, and *S. typhi*). As shown in Table 1, the MIC of the positive control, vancomycin against MSSA was observed at the sixth well (1.56 mg/mL). Meanwhile, the MIC of maggot ES was seen at the fourth well (12.5 mg/mL). In contrast, the MIC of vancomycin against MRSA was higher (6.25 mg/mL), but the maggot ES had the same inhibitory effect against MRSA at a MIC equivalent to 12.5 mg/mL.

**Table 1:** MICs of standard antibiotics and larval extracts against bacteria

Microorganism	Antibiotics (100 mg mL^−1^)	*W. nuba* larval extract (200 mg mL^−1^)
Gram-positive bacteria		
MSSA	Vancomycin (1.56)	12.5 mg mL^−1^
MRSA	Vancomycin (6.25)	12.5 mg mL^−1^
Gram-negative bacteria		
*P. aeruginosa*	Gentamycin (3.12)	12.5 mg mL^−1^
*E. coli*	Gentamycin (1.56)	25 mg mL^−1^
*S. typhi*	Ciprofloxacin (3.12)	25 mg mL^−1^

*MSSA*, methicillin-sensitive *S. aureus*; *MRSA*, methicillin-resistant *S. aureus*; *P. aeruginosa*, *Pseudomonas aeruginosa*; *E. coli*, *Escherichia coli*; *S. typhi*, *Salmonella typhi*

Regarding gram-negative bacteria, the MIC of gentamycin against *P. aeruginosa* was equal to 3.12 mg/mL but against *E. coli* was 1.56 mg/mL. However, MICs of maggot ES were higher against *P. aeruginosa* and *E. coli* (12.5 mg/mL and 25 mg/mL, respectively). The MIC of ciprofloxacin against *S. typhi* was 3.12 mg/mL, but the MIC of maggot ES against *S. typhi* was similar to that observed in *E. coli* (25 mg/mL).

When aliquots were removed from the corresponding MIC wells of selected antibiotics (vancomycin, gentamycin, and ciprofloxacin), no bacterial growth was observed for all plates of BHIA. However, aliquots of all tested bacteria displayed growth on BHIA plates after they were removed from the MIC wells of *W. nuba* ES.

### Quantification of antibacterial activity of ES using MIC

**Fig. 1:**
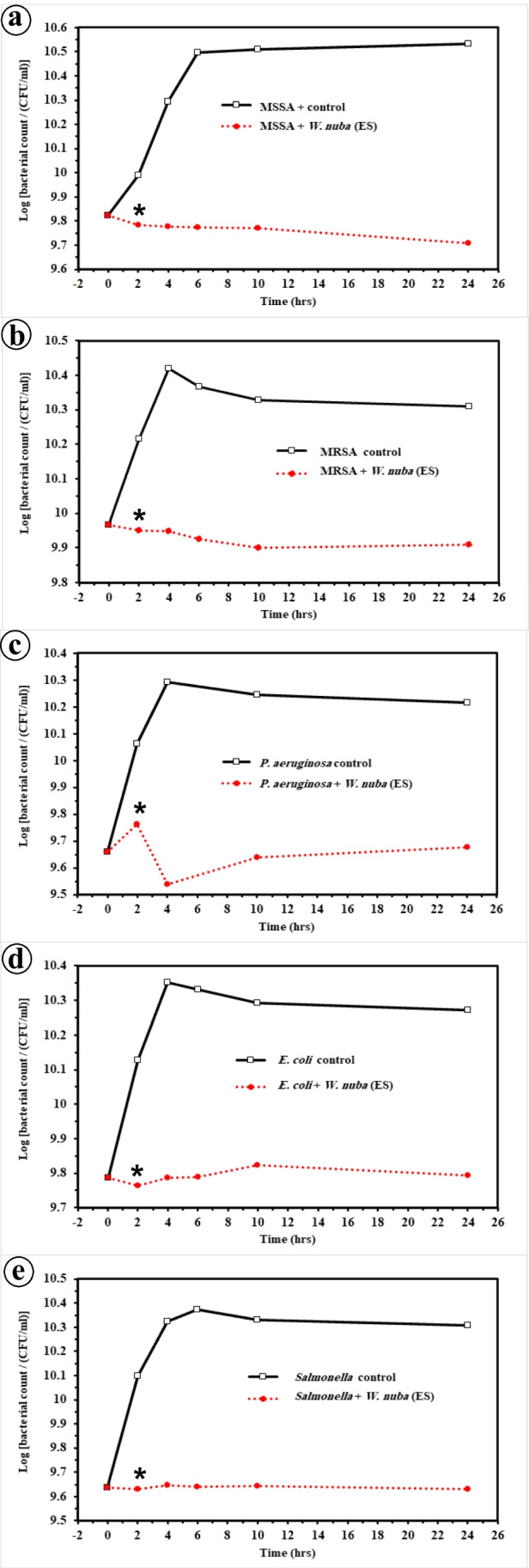
Mean viable counts of **a** methicillin-sensitive *Staphylococcus aureus*, **b** methicillin-resistant *Staphylococcus aureus*, **c** *P. aeruginosa*, **d** *E. coli*, and **e** *S. typhi*, in control and with the addition of *W. nuba* ES at times 0, 2, 4, 6, 10 and 24 h. * = significant versus control starting from 2 h till end of experiment

In the control experiment, the dynamic growth of the five bacterial species tested were similar over a 24 h period (*p* > 0.05), where there was no lag period and the growth began early and rapidly entered the log phase to follow a normal bacterial growth curve (Fig. 1a-e). In the treated experiment, the ES of *W. nuba* displayed bacteriostatic activity against the five bacterial species with varying levels. All the bacterial species failed to recover by the end of 24 h of incubation (Fig. 1a-e). Moreover, there was a highly significant difference in the percentage of surviving bacteria between controls and treated samples with all the bacterial species tested (*p* < 0.001) throughout the experiment. The only exception was observed with MSSA where there was no significant difference between the control and treated samples in the bacterial count after 2 h (*p* = 0.21).

### Gram-positive bacteria

#### MSSA

*Wohlfahrtia nuba* extract exhibited inhibition activity and reduction in the bacterial count of MSSA starting from 2 h (*p* < 0.01) and continued through the experiment. The highest reduction in the bacterial count (80%) was observed at the end of experiment compared with the control (Fig. 1a).

#### MRSA

The dynamics of growth of MRSA following incubation with *W. nuba* extract showed good inhibition of growth throughout the experiment (*p* < 0.01). The reduction of bacterial cells reached 63% comparing with the control within 10 h. A plateau in the number of bacterial cells was observed during the period between 10 and 24 h (Fig. 1b).

### Gram-negative bacteria

#### *Pseudomonas aeruginosa*

*Wohlfahrtia nuba* extract allowed some growth of *P. aeruginosa* for the first 2 h (*p* < 0.01). Then ES reduced the initial count of the bacteria by 24% over a 4 h period, where the bacterial count became about 83% less than the control. Although few growths were observed from 10 h to the end of the experiment, the bacterial count still decreased by 71% compared with the control (Fig. 1c).

#### *Escherichia coli*

The result of the growth curve of *E. coli* following incubation with the extract displayed fluctuation between few decreases and increases in the bacterial count compared with the original population throughout the experiment (*p* < 0.01). At the end of the experimental period (24 h), the reduction in the bacterial count was 67% compared with the control (Fig. 1d).

#### *Salmonella typhi*

Although the ES of *W. nuba* failed to decrease the number of *S. typhi* cells than the initial population, no growth was observed during the time of the experiment (*p* < 0.01). The difference in the bacterial count between treated and control samples was about 79% at the end of experimental period (Fig. 1e).

It is important to note that the selected antibiotics showed bactericidal activity against the tested bacteria within 2 h.

### Quantification of antibacterial activity of ES using different doses at a concentration (200 mg/mL)

Table 2 shows the effect of two different doses, 100 μL and 300 μL, at a concentration of 200 mg/mL against the five bacterial species tested. The results revealed that there was a significantly higher bacterial count in the control experiment compared to the ES experiment (*p* < 0.001) after 18 h. Furthermore, both doses exhibited bactericidal activity against MRSA and *P. aeruginosa*, where no bacterial growth was observed after 18 h of incubation. Additionally, the higher dose 300 μL had a significant effect on the growth of MSSA and *S. typhi* (*p* < 0.001), where the lower dose was less effective at inhibiting bacterial growth compared to the higher dose 300 μL. However, there was no significant difference between the antibacterial activity of ES at 100 and 300 μL against *E. coli* (*p* = 0.81).

**Table 2:** Antibacterial activity of ES in the colony-forming unit (CFU) assay

	Gram-positive bacteria				Gram-negative bacteria					
Excretions/secretions (ES)	MSSA (CFU mm^−1^)		MRSA (CFU mm^−1^)		*P. aeruginosa* (CFU mm^−1^)		*E. coli* (CFU mm^−1^)		*S. typhi* (CFU mm^−1^)	
	At 0 hr	At 18 hrs	At 0 hr	At 18 hrs	At 0 hr	At 18 hrs	At 0 hr	At 18 hrs	At 0 hr	At 18 hrs
Control	4.95 × 10^7^	2.45 × 10^8^	8.30 × 10^7^	1.88 × 10^8^	3.70 × 10^7^	1.37 × 10^8^	8.75 × 10^7^	2.75 × 10^8^	5.00 × 10^7^	2.41 × 10^8^
100 μL	4.95 × 10^7^	4.01 × 10^7a,b^	8.30 × 10^7^	0.00	3.70 × 10^7^	0.00	8.75 × 10^7^	2.98 × 10^7b^	5.00 × 10^7^	3.77 × 10^7a,b^
300 μL	4.95 × 10^7^	0.88 × 10^7a,b^	8.30 × 10^7^	0.00	3.70 × 10^7^	0.00	8.75 × 10^7^	3.12 × 10^7b^	5.00 × 10^7^	1.59 × 10^7a,b^

*MSSA*, methicillin-sensitive *Staph aureus*; *MRSA*, methicillin-resistant *S. aureus*; *P. aeruginosa*, *Pseudomonas aeruginosa*; *E. coli*, *Escherichia coli*; *S. typhi*, *Salmonella typhi*; *CFU*, colony-forming unit; *hr*, hour; *mm*, millimeter. Data were analyzed with chi-square test

^a^Letter means significant difference in the same column at (*p* < 0.05)

^b^Letter means significant difference with control in the same column at (*p* < 0.05)

### Antibacterial activity of *W. nuba* maggot ES using disc diffusion assay

**Fig. 2:**
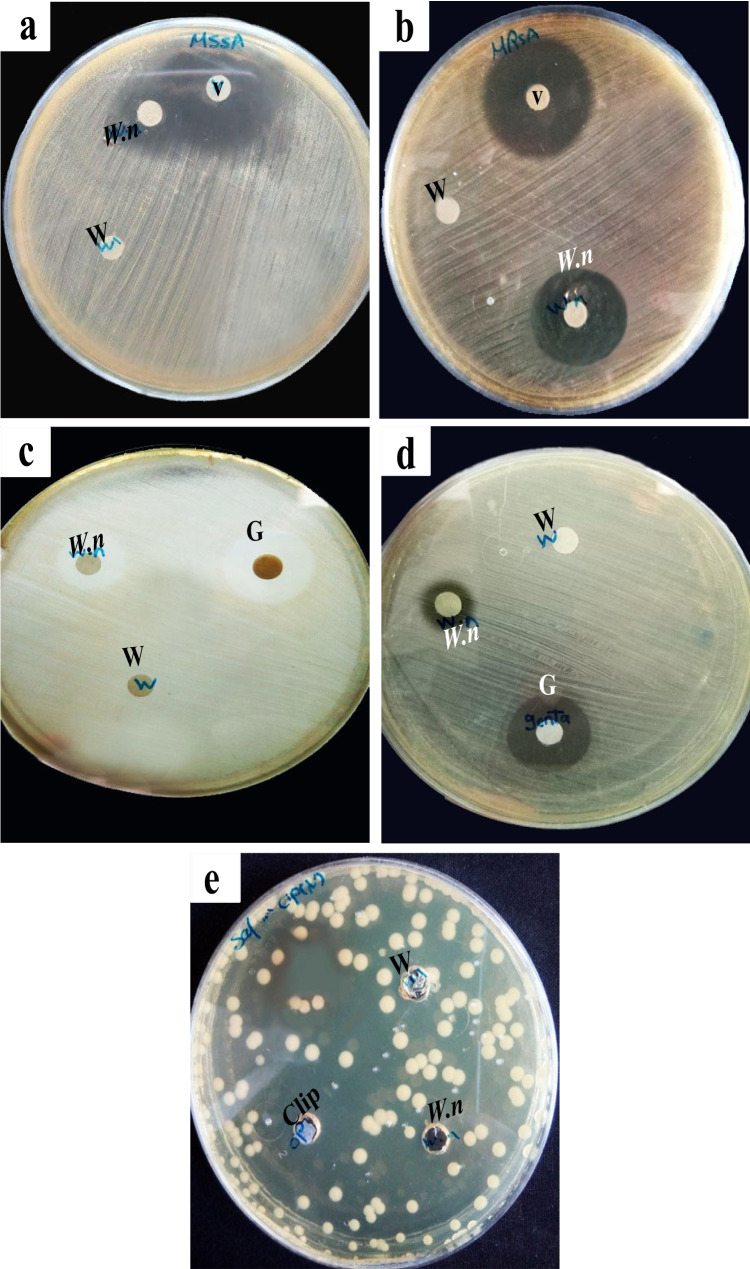
Results of antibacterial activity of *W. nuba* (ES) showing zones of inhibition for **a** methicillin-sensitive *Staphylococcus aureus*, **b** methicillin-resistant *Staphylococcus aureus*, **c** *P. aeruginosa*, **d** *E. coli*, **e** *S. typhi*. *W.n*, *W. nuba* larval extract; V, vancomycin; G, gentamycin; Cip, ciprofloxacin; and W, distilled water

The results of the antibacterial activity of *W. nuba* maggot ES using disc diffusion are presented in Table 3 and Fig. 2a,b,c,d,e. ANOVA showed that there were significant differences in the diameter of inhibitory zones of MSSA, *E. coli*, and *S. typhi* between standard antibiotics and maggot ES (*p <* 0.05). However, no significant differences were recorded between antibiotics in both *P. aeruginosa* and MRSA. The results indicated that disc diffusion failed to show any antibacterial activity of maggot ES against gram-positive bacteria by using a low dose (20 μL) of ES at 200 mg/mL concentration, where no zones were observed around the discs. However, a high dose (50 μL) of ES showed high potency against MSSA and MRSA as indicated by the diameter of inhibition zones (19.6 ± 0.33 and 20.0 ± 1.15 mm, respectively). Regarding gram-negative bacteria, the inhibition zone diameter of *P. aeruginosa* and *E. coli* for (20 μL) ES showed significant differences between them (*p* < 0.05). Meanwhile, only the dose of 50 μL ES provided activity that inhibited the growth of *S. typhi.*

**Table 3:** Antimicrobial activity of excretions/secretions (ES) of *W. nuba* larvae evaluated by disc diffusion method

	Inhibition zone (mean ± SE by mm)		
Microorganism	Antibiotics		Excretions/secretions (ES)
Gram-positive bacteria			
MSSA	VA-30 mcg	28.67 ± 0.67^c^	19.67 ± 0.33
MRSA	VA-30 mcg	24.00 ± 1.00	20.00 ± 1.15
Gram-negative bacteria			
*P. aeruginosa*	CN-10 mcg	17.33 ± 1.20	14.67 ± 0.88^a,b^
*E. coli*	CN-10 mcg	17.00 ± 1.53^c^	11.33 ± 0.67^a,b^
*S. typhi*	CIP-5 mcg	31.33 ± 0.88^c^	14.00 ± 0.58^b^

*MSSA*, methicillin-sensitive *Staph aureus*; *MRSA*, methicillin-resistant *S. aureus*; *P. aeruginosa*, *Pseudomonas aeruginosa*; *E. coli*, *Escherichia coli*; *S. typhi*, *Salmonella typhi*; *VA-30 mcg*, vancomycin; *CN-10 mcg*, gentamycin; *CIP-5 mcg*, ciprofloxacin. Data were analyzed with one-way ANOVA

^a^Letter means significant difference in the same column (*p* < 0.05)

^b^Letter means significant difference (*p* < 0.05) in inhibition zone with G + bacteria in the same column

^c^Letter means significant difference in zone of inhibition between ES and corresponding antibiotic at *p* < 0.05

According to Zhou et al. [24], the detection criteria for growth inhibition were categorized as follows: an inhibition zone diameter ≤ 10 mm for low sensitivity, 10–14 mm for moderate sensitivity, 14–19 mm for sensitivity, and ≥ 19 mm for high sensitivity. Therefore, the present results suggest that the ES extract of *W. nuba* is highly sensitive against gram-positive bacteria, sensitive against *P. aeruginosa* and *E. coli*, and moderately sensitive against *S. typhi*.

### Evaluation of the synergistic effect between the larval extract and antibiotics

**Fig. 3:**
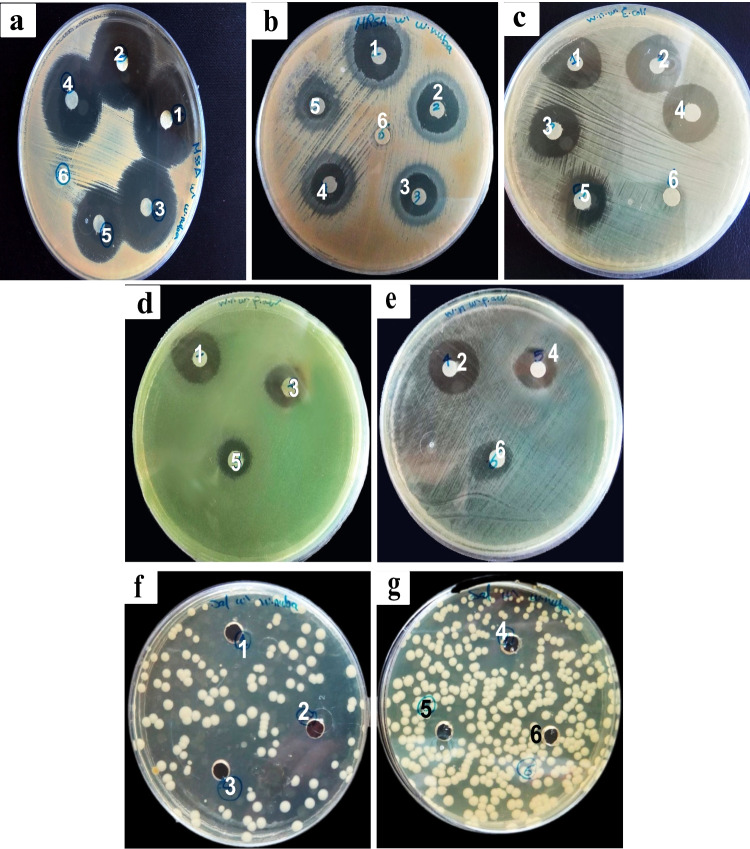
Effect of each antibiotic alone and in combination with *W. nuba* larval extract on growth of gram-positive and gram-negative bacteria. Vancomycin: **a** methicillin-sensitive *Staphylococcus aureus*, and **b** methicillin-resistant *Staphylococcus aureus*; Gentamycin: **c** *E. coli*, and **d**, **e** *P. aeruginosa*; Ciprofloxacin: *S. typhi* (**f**, **g**). 1 = 100% AB; 2 = 80% AB+20% ES; 3 = 60% AB+40% ES; 4 = 40% AB+60% ES; 5 = 20% AB+80% ES; 6 = 100% ES

Results of a combination of vancomycin, gentamycin, and ciprofloxacin with maggot ES showed a synergistic effect against the entire studied bacterial species using disc diffusion assay (Table 4; Fig. 3a–g). However, the largest zone of inhibition against any bacterial species tested was observed with the antibiotic alone.

**Table 4:** Effect of combination between ES and antibiotics on the viability of bacteria

			Inhibition zone (mean ± SE by mm)		
Concentration (AB/ES)	MSSA/VA	MRSA/VA	*P. aeruginosa*/CN	*E. coli*/CN	*S. typhi*/CIP
100% AB	29.33 ± 0.67	23.67 ± 0.33	19.33 ± 0.33	20.67 ± 0.33	29.00 ± 1.15
80% AB+20% ES	29.00 ± 0.58	22.33 ± 0.33^a^	18.67 ± 0.33^b^	19.67 ± 0.33^b^	29.00 ± 1.15^a^
60% AB+40% ES	28.67 ± 0.33	21.00 ± 0.58^a^	15.33 ± 0.33^a,b^	19.67 ± 0.33^b^	25.00 ± 0.58^a^
40% AB+60% ES	28.67 ± 0.33	21.00 ± 0.58^a^	14.33 ± 0.33^a^	16.67 ± 0.33^a,b^	19.00 ± 0.58^a^
20% AB+80% ES	23.67 ± 0.33^a^	18.67 ± 0.33^a^	12.67 ± 0.33^a^	14.67 ± 0.33^a,b^	14.00 ± 0.58^a^
100% ES	NZ	NZ	13.00 ± 0.58	10.00 ± 0.58	NZ

*MSSA*, methicillin-sensitive *Staph aureus*; *MRSA*, methicillin-resistant *S. aureus*; *P. aeruginosa*, *Pseudomonas aeruginosa*; *E. coli*, *Escherichia coli*; *S. typhi*, *Salmonella typhi*; *AB*, antibiotic tested; *ES*, maggot extract; *NZ*, no zone of inhibition. Data were analyzed with one-way ANOVA

^a^Letter means that combination is significantly different with 100% AB in the same column at (*p* < 0.05)

^b^Letter means that combination is significantly different with 100% ES in the same column at (*p* < 0.05)

Regarding gram-positive bacteria, vancomycin in combination with ES at a percentage of 80% VA + 20% ES against MSSA did not show any reduction in the diameter of the inhibition zone (29 ± 0.58 mm) compared to the antibiotic alone (29.33 ± 0.67 mm). Significant reduction in the diameter of the inhibition zone of MSSA was observed only in combination with 20% VA and 80% ES compared to antibiotic alone (*p* < 0.001). However, all combinations of vancomycin and ES showed significant alternations in the diameter of the inhibition zone against MRSA (*p* < 0.01).

Regarding gram-negative bacteria, a combination of maggot ES with gentamycin was less effective against *E. coli* than against *P. aeruginosa*, where significant decreases in the diameters of the inhibition zone were recorded starting from 60% CN + 40% ES with *P. aeruginosa*, and from 40% CN + 60% ES with *E. coli.* Ciprofloxacin in combination with ES at a percentage of 80% CIP + 20% ES showed equal diameters of inhibition zones (29 ± 1.15 mm) against *S. typhi* as ciprofloxacin*.* The remaining combinations exhibited significant reduction in the diameter of the inhibition zone (*p* < 0.001).

It is interesting to note that ES alone with a dose of 25 μL (at a concentration of 200 mg/mL) did not have any antibacterial activity against MSSA, MRSA, and *S. typhi*.

## Discussion

With the increase of antibiotic-resistant pathogens, it is important to look for newer substances that have antibacterial activity. Previous studies have shown that maggot therapy is successful in the treatment of infected wounds [27, 28]. The present study investigated the influence of the ES of the *W. nuba* maggot on the growth inhibition of both gram-negative and gram-positive bacteria. Although the ES of *W. nuba* was collected from non-bacterial treated maggots reared on raw beef, the results of all assays applied in this study indicated that the *W. nuba* maggot ES has an inhibitory effect against all tested bacteria in vitro. Our results confirmed the previous results of Kerridge et al. [9] who reported that many of the maggots’ antibacterial compounds are constitutively expressed and do not require induction [29], especially those synthesized in secretory cells [30]. In another study, Kaihanfar et al. [31] reported that the ES obtained from bacteria-exposed maggots, of *Lucilia sericata*, had a greater ability to inhibit bacterial growth compared to non-bacterial exposed maggots.

Resazurin-based turbidimetric assay was used in this study to determine the MICs of the larval ES of *W. nuba* against a range of pathogenic bacteria. Teh et al. [22] reported that the resazurin method could be considered a rapid, simple, reliable, sensitive, and efficient antibacterial assay. They demonstrated that the MICs of the *Lucilia cuprina* larval extract obtained from resazurin assay were relatively higher than those determined by the traditional turbidimetric (TB) assay [32]. Furthermore, conventional TB assay was less sensitive to bacterial growth than resazurin, as TB assay was sensitive in case of 50% bacterial growth. In contrast, resazurin assay was sensitive with the presence of only 80 cells of bacteria, which led to the reduction of resazurin to resorufin and the change of its color from blue to pink.

In our study, despite the inhibitory effect of the larval ES of *W. nuba* against all the tested bacteria, the MICs of *W. nuba* larval ES were higher (25 mg/mL) against *E. coli* and *S. typhi* than the MICs against MSSA, MRSA, and *P. aeruginosa* (12.5 mg/mL) using the resazurin assay. This indicates that the larval ES was more effective at inhibiting the growth of MSSA, MRSA, and *P. aeruginosa*. Thus, we conclude that the *W. nuba* ES has an inhibitory effect against all the tested bacteria with varying MICs that were lower than those previously determined by Teh et al. [22]. In their study, Teh et al. [22] applied the same assay and demonstrated that the *Lucilia cuprina* larval extract had an inhibitory effect against four tested bacteria, namely, *S. aureus*, MRSA, *P. aeruginosa*, and *E. coli*, with a MIC of 100 mg/mL. However, the larval extract of *Sarcophaga peregina* and *Musca domestica* had an inhibitory effect against MRSA only with a MIC of 100 mg/mL. Moreover, according to the results of El-Bassiony and Stoffolano [18], the larval ES of *S. argyrostoma* was more effective against gram-negative bacteria, where the MIC of 0.125 mg/mL was significantly potent towards *P. aeruginosa* and *E. coli*. High MICs were required to inhibit the growth of *Staph aureus* (0.25 mg/mL) and *Bacillus subtilis* (0.5 mg/mL), using conventional TB assay.

Regarding antibacterial properties, the incapability of bacterial cells to resume growth on BHIA after transfer from the MIC wells revealed that the standard antibiotics were bactericidal against all the tested bacteria at the corresponding MICs. However, the larval extract of *W. nuba* was unable to inhibit the growth of all the tested bacteria at the corresponding MICs, and this suggested that the larval extract of *W. nuba* exerts a bacteriostatic effect. Nevertheless, the fact that the *W. nuba* extract did not maintain an inhibitory effect on bacterial growth during the experimental period does not reduce its potential activity as an antibacterial agent. Several experiments demonstrated that ES are secreted continuously through the maggot life cycle during maggot therapy [17]. However, in the present in vitro experiment, ES was added only once to the bacteria. Therefore, despite the short duration of the antibacterial activity of ES, it would still be effective against bacteria in vitro [16]. It is worth noting that the MICs of the maggot extract were higher than the MICs of standard antibiotics; nevertheless, these antibiotics consisted of purified active compounds as compared to the crude extract of the fly maggots. It is expected that a smaller amount of maggot extract would exert similar antibacterial activity if the purified form of maggot extract could be manufactured. This could be used in the production of an antimicrobial product of natural origin [22].

According to the results of colony-forming units assay, using the MICs of *W. nuba* ES (as determined in the present study) showed a significant inhibitory effect against all the bacterial species tested over a 24 h period. This indicates that the *W. nuba* ES possesses one or more factors capable of inhibiting both gram-positive and gram-negative bacteria in vitro, despite differences between the bacterial cell walls [9, 11]. Similarly, potent antibacterial activity against different species of bacteria has been reported in the ES of some calliphorid and sarcophagid species [14, 17, 18, 33].

Moreover, the larval extract of *W. nuba* caused some decrease in the bacterial numbers of *P. aeruginosa*, *E. coli*, and *S. aureus* for a short period. The inhibitory effect of the *W. nuba* maggot ES against the tested bacteria may be related to the defensive group (low molecular weight peptides) that is found in different insects [9, 34, 35]. The defensive group has a high ability to attach to phospholipids in bacteria, which leads to the formation of channels on the bacterial cytoplasmic membrane that are responsible for the destruction of the bacteria [36]. These peptides give insects their intrinsic ability to fight against several pathogenic bacteria [14] and may be considered a main factor responsible for inhibiting/decreasing bacterial growth in the present study.

Nevertheless, the presence of microflora that secretes antibacterial components such as oligopeptides in the digestive system of maggots [11] may be another factor that led to the present result. Generally, the broad spectrum of antibacterial activity of *W. nuba* against both gram-positive and gram-negative bacteria may be due to the range of bacterial species that the maggots are exposed to in their natural carrion feeding environment or within a wound [9]. Based on the results obtained from the colony-forming units’ assay, it is evident that both higher and lower doses of ES at a concentration of 200 mg/mL showed bactericidal activity against MRSA and *P. aeruginosa* after 18 h. Furthermore, the high dose of ES (300 μL) was more potent against both MSSA and *S. typhi* than the lower dose (100 μL) at the same concentration. However, a higher dose of the ES failed to show any reduction in *E. coli* counts compared with the lower dose after 18 h. The *W. nuba* ES varied in its potency dependent on both the dose and concentration of the ES as well as the bacterial species tested. This was similar to the results of Chaiwong et al. [37] who reported that a higher dose of *Chrysomaya megacephala* ES was more effective in reducing the *E. coli* count than a lower dose. However, the *Chrysomaya megacephala* ES was not found to inhibit both *P. aeruginosa* and *S. aureus*.

Results of the antibacterial activity of the maggot ES of *W. nuba* evaluated by disc diffusion assay revealed that the current dose of larval ES (20 μL) exhibited antibacterial activity towards *P. aeruginosa* and *E. coli*. However, this dose did not show any activity towards the remaining bacteria where no zones of inhibition developed around the discs containing the ES. The higher dose of the larval ES (50 μL) was apparently active towards gram-positive bacteria (MSSA and MRSA) as well as gram-negative bacteria (*S. typhi*) suggesting that 20 μL of the larval ES was considered an insufficient dose and concentration to exert its antibacterial activity. In our study, the ES of *W. nuba* killed MSSA, MRSA, and *S. typhi* in a dose-dependent manner with as little as 50 μL (200 mg/mL) of ES being effective. It is important to note that more *W. nuba* ES is required to inhibit the growth of MSSA, MRSA, and *S. typhi* when it is applied for the treatment of these bacterial infections. Our results confirmed the results of previous studies carried out by Ge et al. [38] where the crude extract of *Lucilia sericata* showed obvious, dose-dependent antibacterial effects against *E. coli*. Moreover, the results of Auza et al. [39] indicated that the antibacterial activity of the extract of black soldier flies increased significantly with the increase of extract concentration, and 325 mg/mL was the most effective concentration for inhibiting the growth of *S. typhimurium*, *E. coli*, and *P. aeruginosa*.

Based on the diameter of the inhibition zone, *W. nuba* ES showed strong inhibition of growth in gram-positive bacteria (MSSA, MRSA). Regarding gram-negative bacteria, the diameter of the inhibitory zone of *P. aeruginosa* increased significantly compared to *E. coli*, indicative of a more potent effect of the ES against *P. aeruginosa.* However, compared with the control antibiotic (ciprofloxacin), the growth of inhibition of *S. typhi* decreased more than two orders of magnitude by applying 50 μL of the ES. The larval ES from *W. nuba* showed a different level of sensitivity to all the tested bacterial species. This may be due to differences in the interaction of the bacterial cell wall components with the active components of the larval *W. nuba* extract [40]. In this regard, Yakovleva et al. [41] reported that the inhibitory concentrations of some regular antibiotics used against *E. coli*, *Klebsiella pneumoniae*, and *Actinobacter baumannii* were more effective by more than two folds compared with the antimicrobial peptide complex of *Calliphora vicina*.

The maggot ES and different antibiotics had a different synergistic ability to inhibit the growth of the tested bacterial species depending on the doses of both the ES and the antibiotics. The effect of the combination of a lower dose of vancomycin and ES was somewhat equivalent to that of a normal dose of vancomycin alone as determined by the overall nearly similar diameter of the inhibition zones. This suggests that the maggot ES acts synergistically with vancomycin against MSSA. Moreover, the addition of ES to vancomycin at a 20% MIC concentration displayed an inhibition zone indicating that it enhances the effect of a standard antibiotic [42]. Therefore, a low dose of vancomycin in combination with the maggot ES could be applied in clinical practice against MSSA to reduce the side effects of the antibiotic.

Nonetheless, gentamycin showed a stronger synergistic effect with the ES of *W. nuba* against *E. coli* than *P. aeruginosa*, where the ES helped enhance the activity of gentamycin at sub-MIC levels (60% and 80% MIC concentration against *E. coli* and *P. aeruginosa*, respectively). Thus, the synergism between gentamycin and ES could be useful in clinical applications. A low concentration of gentamycin is bactericidal in the presence of maggot ES and could decrease the risks of severe gentamycin-related side effects, such as nephrotoxicity and hearing loss, thus providing more patient safety [43]. The current results are consistent with those obtained in previous studies [44, 45], which showed that the *Lucilia sericata* ES could increase the activity of gentamycin against bacteria in vitro at low concentrations.

The combination of the *W. nuba* ES with ciprofloxacin was able to enhance ciprofloxacin activity (at 80% MIC concentration) against *S. typhi* and showed high potency (29 ± 1.15 mm) similar to that of ciprofloxacin alone. Moreover, the addition of the ES to a 20% MIC concentration of ciprofloxacin enabled, to some extent, the inhibition of the development of *S. typhi*. This is consistent with the results of Arora et al. [15] who reported better synergism between the ES of *Lucilia sericata* and ciprofloxacin (at 60% and 80% MIC concentrations) against *S. aureus*. They also reported that antibiotics with a concentration of 100% lost their inhibitory impact on bacterial growth after 6 days, but the larval ES retained its inhibitory effect against bacteria.

One of the effective ways to overcome bacterial resistance is the restoration of antibacterial activity through the synergistic action of antibacterial compounds from natural agents [46]. This is supported by Chernysh et al. [47] who reported that the antimicrobial peptide complex of *Calliphora vicina* acted as a synergist for beta-lactam, aminoglycoside, glycopeptide, and cephalosporin antibiotics. Generally, the use of antibiotics in combination with larval extract from the start may aid the reduction of the development of resistance in bacterial species [48].

## Conclusion

The present study shows for the first time the bacteriostatic activity of the ES of the maggot of *W. nuba* against *S. aureus*, *E. coli* and *S. typhi*, and its bactericidal activity against MRSA and *P. aeruginosa*. *W. nuba* ES can be used in combination with a low concentration of classic antibiotics to reduce the risks of antibiotic-related side effects. Overall, this study provides a new source of antimicrobial compounds to a broad range of bacterial species, in addition to the other previously investigated carrion-feeding fly species.

## Supplementary information

ESM 1 (PNG 2560 kb)

High resolution image (TIF 2280 kb)

ESM 2 (PNG 5489 kb)

High resolution image (TIF 4616 kb)

ESM 3 (PNG 5260 kb)

High resolution image (TIF 4432 kb)

## References

[1] Cezard C, Pires SV, Mullie C, Sonnet P (2011) Antimicrobial peptides: a review. In: Mendez-Vilas (ed) Science against microbial pathogens: communicating current research and technological advances. FORMATEX, pp 926-937

[2] Davies J, Davies D (2010). Origins and evolution of antibiotic resistance. Microbiol Mol Biol Rev.

[3] Meimeti E, Lykoudi E, Arapostathi E, Tsagkarakis AE, Papavramidou N, Rallis M, Galanis I (2021). Maggot debridement therapy: from the battlefields and soldiers to today’s clinical trials. Int J Caring Sci.

[4] Malekian A, Esmaeeli Djavid G, Akbarzadeh K, Azam M, Rassi Y, Rafinejab J, Foroushani AR, Farhoud A, Bakhtiary R, Totonchi M (2019). Efficacy of maggot therapy on *Staphylococcus aureus* and *Pseudomonas aeruginosa* in diabetic foot ulcers: a randomized controlled trial. J Wound Ostomy Cont Nurs.

[5] Mylonakis EM, Kalli I, Rallis ST (2016). Canine parvoviral enteritis: an update on the clinical diagnosis, treatment, and prevention. Veterinary J Vet Med Res.

[6] Gordon YJ, Romanowski EG, McDermott AM (2005). A review of antimicrobial peptides and their therapeutic potential as antiinfective drugs. Curr Eye Res.

[7] Bahar AA, Ren D (2013). Antimicrobial peptides. J Pharm.

[8] Chernysh S, Gordya N, Suborova T (2015). Insect antimicrobial peptide complexes prevent resistance development in bacteria. PLoS One.

[9] Kerridge A, Lappin-Scott H, Stevens JR (2005). Antibacterial properties of larval secretions of the blowfly, *Lucilia sericata*. Med Vet Entomol.

[10] Thomas S, Andrews AM, Hay NP, Bourgoise S (1999). The anti-microbial activity of maggot secretions: results of a preliminary study. J Tissue Viability.

[11] Bexfield A, Nigam Y, Thomas S, Ratcliffe NA (2004). Detection and partial characterization of two antibacterial factors from the excretions/secretions of the medicinal maggot *Lucilia sericata* and their activity against methicillin-resistant *Staphylococcus aureus* (MRSA). Microbes Infect.

[12] Huberman L, Gollop N, Mumcuoglu KY, Block C, Galun R (2007). Antibacterial properties of whole-body extracts and haemoloymph of *Lucilia sericata* maggots. J Wound Care.

[13] Jaklic D, Lapanje A, Zupancic K, Smrke D, Gunde-Cimerman N (2008). Selective antimicrobial activity of maggots against pathogenic bacteria. J Med Microbiol.

[14] Arora S, Lim CS, Baptista C (2010). Antibacterial activity of *Lucilia cuprina* maggot extracts and its extraction techniques. Int J Integr Biol.

[15] Arora S, Baptista C, Lim CS (2011). Maggot metabolites and their combinatory effects with antibiotic on *Staphylococcus aureus*. Ann Clin Microbiol Antimicrob.

[16] Barnes KM, Gennard DE, Dixon RA (2010). An assessment of the antibacterial activity in larval excretion/secretion of four species of insects recorded in association with corpses, using *Lucilia sericata* Meigen as the marker species. Bull Entomol Res.

[17] Masiero FS, Aquino MFK, Nassu MP, Pereira DIB, Leite DS, Thyssen PJ (2017). First record of larval secretions of *Cochliomyia macellaria* (Fabricius, 1775) (Diptera: Calliphoridae) inhibiting the growth of *Staphylococcus aureu*s and *Pseudomonas aeruginosa*. Neotrop Entomol.

[18] El-Bassiony GM, Stoffolano JG (2016). In vitro antimicrobial activity of maggot excretions/secretions of *Sarcophaga* (Liopygia) *argyrostoma* (Robineau-Desvoidy). Afr J Microbiol Res.

[19] Moneer SA, Kotb MH, Ahmed ZI, Ahmed IA, Hasballah MMM (2019). Antimicrobial and antiviral activity of *Lucilia sericata*, *Chrysomya albiceps* (Diptera: Calliphoridae) and *Musca domestica* (Diptera: Muscidae) whole body extract. Egypt Acad J Biolog Sci.

[20] Sherman RA, Hall MJ, Thomas S (2000). Medicinal maggots: an ancient remedy for some contemporary afflictions. Annu Rev Entomol.

[21] Daeschlein G, Mumcuoglu KY, Assadian O, Hoffmeister B, Kramer A (2007). In vitro antibacterial activity of *Lucilia sericata* maggot secretions. Skin Pharmacol Physiol.

[22] Teh CH, Nazni WA, Nurulhusna AH, Norazah A, Lee HL (2017). Determination of antibacterial activity and minimum inhibitory concentration of larval extract of fly via resazurin-based turbidometric assay. BMC Microbiol.

[23] Okuma K, Iwakawa K, Turnidge JD, Grubb WB, Bell JM, O’Brien FG (2002). Dissemination of new methicillin-resistant *Staphylococcus aureus* clones in the community. J Clin Microbiol.

[24] Zhou G, Wang J, Zhu X, Wu Y, Gao M, Shen H (2014). Induction of maggot antimicrobial peptides and treatment effect in *Salmonella pullorum*-infected chickens. J Appl Poult Res.

[25] CLSI (2020) Performance standards for antimicrobial susceptibility testing, 30th ed, CLSI M100-ED30

[26] Bauer AW, Kirby WM, Sherris C, Turck M (1966). Antibiotic susceptibility testing by a standardized single disk method. Am J Clin Pathol.

[27] Grassberger M (2002). An historical review of the use of maggots in wound therapy. NTM.

[28] Jz W, Wang S, Zhao G, Wang Z, Lineawave WC, Zhang F (2006). Treatment of infected wounds with maggot therapy after replantation. J Reconstr Microsurg.

[29] Huberman L, Gollop N, Mumcuoglu KY, Breuer E, Bhusare SR, Shai Y, Galun R (2007). Antibacterial substances of low molecular weight isolated from the blowfly. Lucilia sericata. Med Vet Entomol.

[30] Hancock REW, Diamond G (2000). The role of antimicrobial peptides in innate host defenses. Trends Microbiol.

[31] Kaihanfar M, Momeni-Moghaddam M, Moghaddam MJM, Hajar T, Pak VD, Bidi JO (2018). Investigation of antimicrobial effects of treated *Lucilia sericata* larvae extract on bacteria. Iran J Microbiol.

[32] Teh CH, Nazni WA, Lee HL, Fairuz A, Tan SB, Sofian-Azirun M (2013). In vitro antibacterial activity and physicochemical properties of a crude methanol extract of the larvae of the blow fly *Lucilia cuprina*. Med Vet Entomol.

[33] Ratcliffe NA, Vieira CS, Mendonça PM, Caetano RL, Queiroz MMDC (2015). Detection and preliminary physico-chemical properties of antimicrobial components in the native excretions/secretions of three species of *Chrysomya* (Diptera, Calliphoridae) in Brazil. Acta Trop.

[34] Bulet P, Hetru C, Dimarcq JL, Hoffmann D (1999). Antimicrobial peptides in insects; structure and function. Dev Comp Immunol.

[35] Seufi AM, El-Bassiony GM, Ibrahim SS (2009). Purification and characterization of two antimicrobial peptides from bacterial-challenged haemolymph of *Bombyx mori* larva. Egypt Acad J Biol Sci.

[36] Yi HY, Chowdhury M, Huang YD, Yu XQ (2014). Insect antimicrobial peptides and their applications. Appl Microbiol Biotechnol.

[37] Chaiwong T, Srivoramas T, Sebsumran P, Panya M, Wanram S, Panomket P (2016). Antibacterial activity of excretions-secretions from *Chrysomya megacephala* against *Escherichia coli*. J Med Assoc Thai.

[38] Ge QS, Zhang HM, Liu X, Wang SY, Lv DC, Li XD (2015). Crude extract of maggots: antibacterial effects against *Escherichia coli*, underlying mechanisms, separation and purification. World J Gastroenterol.

[39] Auza FA, Purwanti S, Syamsu JA, Natsir A (2020). Antibacterial activities of black soldier flies (*Hermetia illucens. l*) extract towards the growth of Salmonella typhimurium, E. coli and Pseudomonas aeruginosa. IOP Conf Series Environ Earth Sci.

[40] Choi YW, Kim YJ, Lee SC, Hong JK, Hwang BK (2007). Hydrogen peroxide generation by the pepper extracellular peroxidase CaPO2 activates local and systemic cell death and defense response to bacterial pathogens. Plant Physiol.

[41] Yakovleva AY, Kruglikovaa AA, Chernysha SI (2019). Calliphoridae flies in medical biotechnology. Entomol Rev.

[42] Rakholiya K, Chanda S (2012). In vitro interaction of certain antimicrobial agents in combination with plant extracts against some pathogenic bacterial strains. Asian Pac J Trop Biomed.

[43] Cazander G, van de Veerdonk MC, Vandenbroucke-Grauls CM, Schreurs MWJ, Jukema GN (2010). Maggot excretions inhibit biofilm formation on biomaterials. Clin Orthop Relat Res.

[44] Van der Plas MJA, Jukema GN, Wai S-W, Dogterom-Ballering HCM (2008). Maggot excretions/secretions are differentially effective against biofilms of *Staphylococcus aureus* and *Pseudomonas aeruginosa*. J Antimicrob Chemother.

[45] Cazander G, van Veen KE, Bernards AT, Jukema GN (2009). Do maggots have an influence on bacterial growth? A study on the susceptibility of strains of six different bacterial species to maggots of *Lucilia sericata* and their excretions/secretions. J Tissue Viability.

[46] Stefanovic O, Stankovic MS, Comic L (2011). In vitro antibacterial efficacy of *Clinopodium vulgare* L. extracts and their synergistic interaction with antibiotics. J Med Plant Res.

[47] Chernysh S, Gordya N, Tulin D, Yakovlev A (2018). Biofilm infections between Scylla and Charybdis: interplay of host antimicrobial peptides and antibiotics. Infect Drug Resist.

[48] Cottarel G, Wierzbowski J (2007). Combination drugs, an emerging option for antibacterial therapy. Trends Biotechnol.

