# Supplementary figures and images for: In vitro antibacterial activity and synergetic effect of crude extract of the Wohlfahrtia nuba (Diptera: Sarcophagidae) flesh fly larvae
Source: Braz J Microbiol. 2023 Jun 20;54(3):1373–85. doi: 10.1007/s42770-023-01024-z (PMC10484856; doi:10.1007/s42770-023-01024-z)

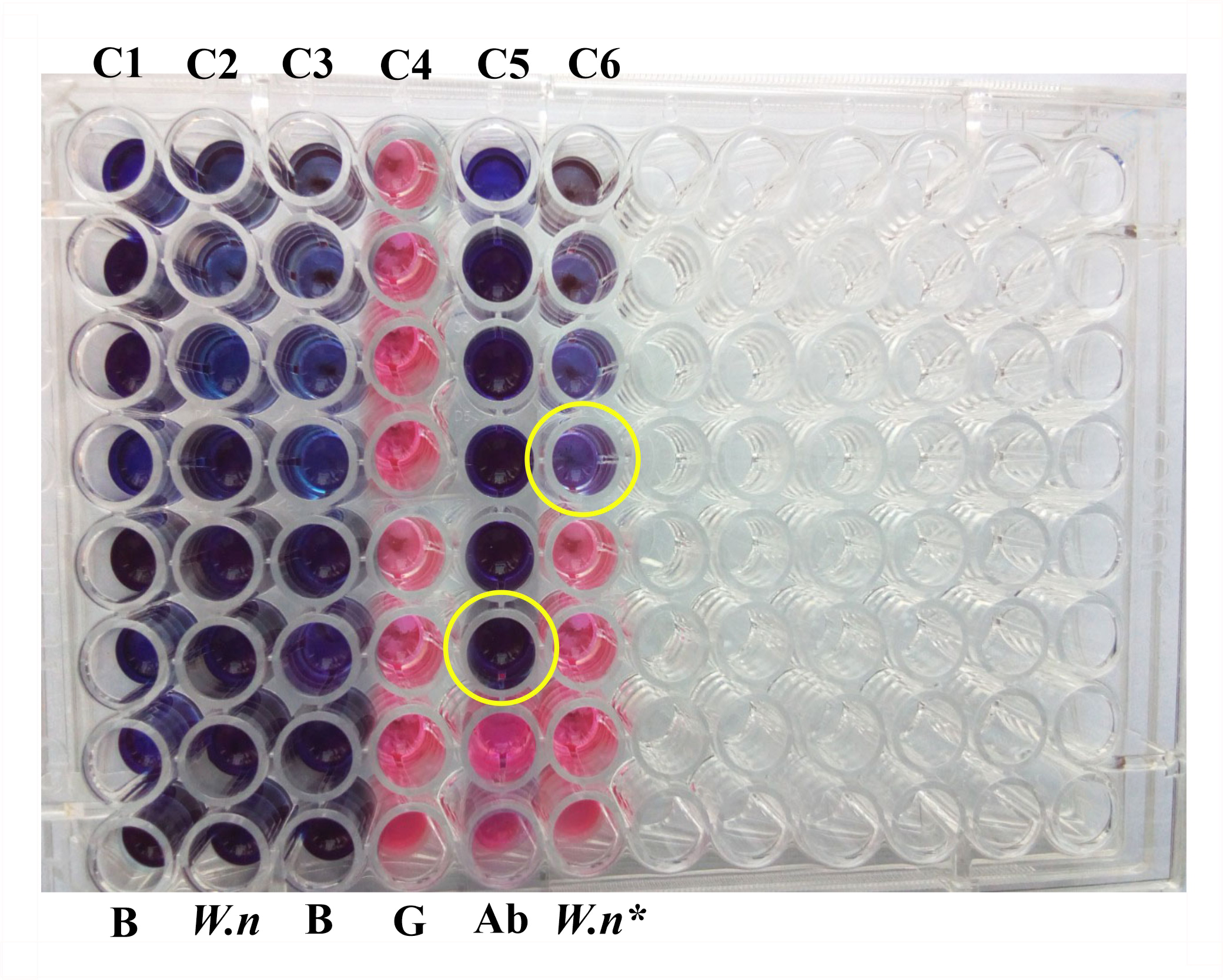

Supplement: Supplementary file 1 — (PNG 2560 kb) [file 42770_2023_1024_Fig4_ESM.png]

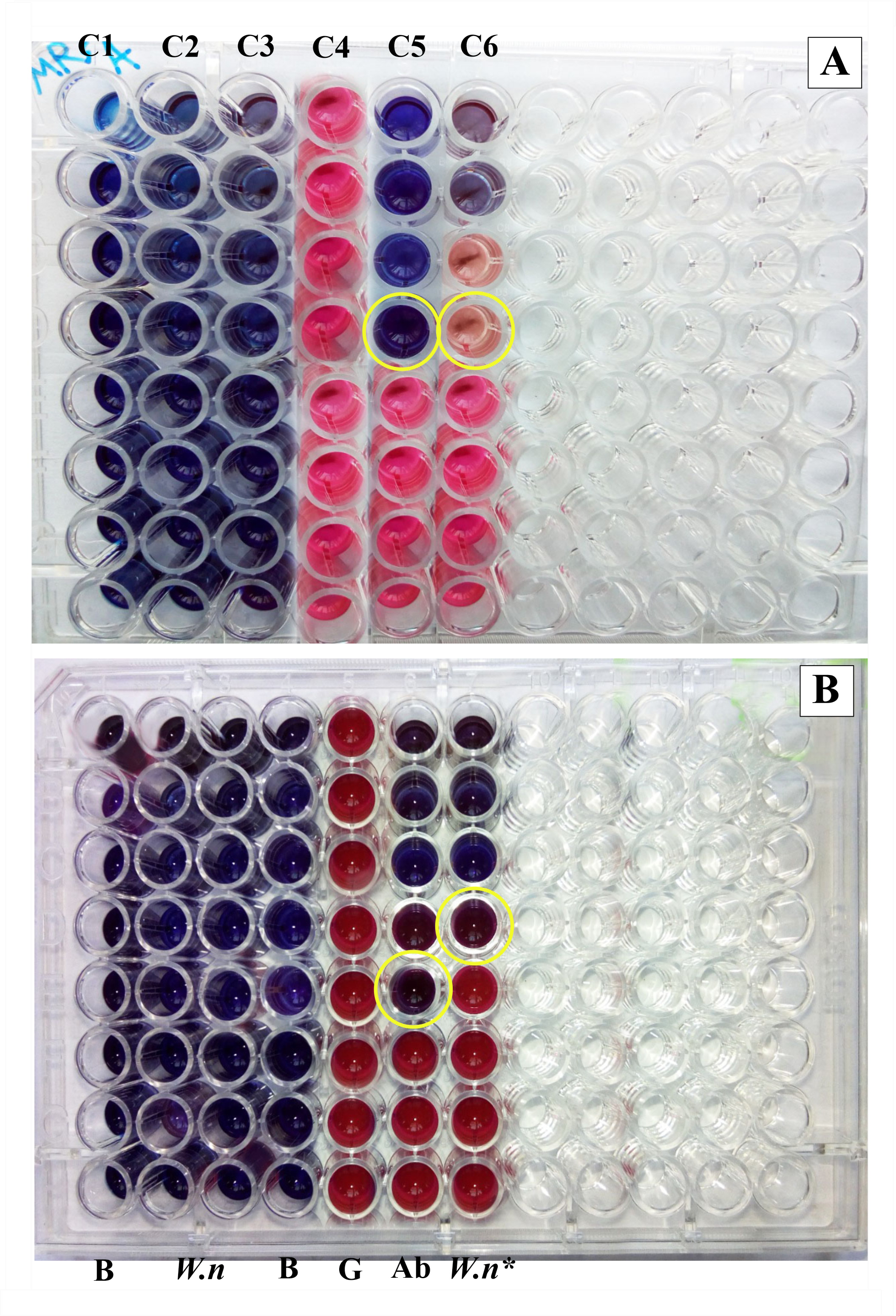

Supplement: Supplementary file 3 — (PNG 5489 kb) [file 42770_2023_1024_Fig5_ESM.png]

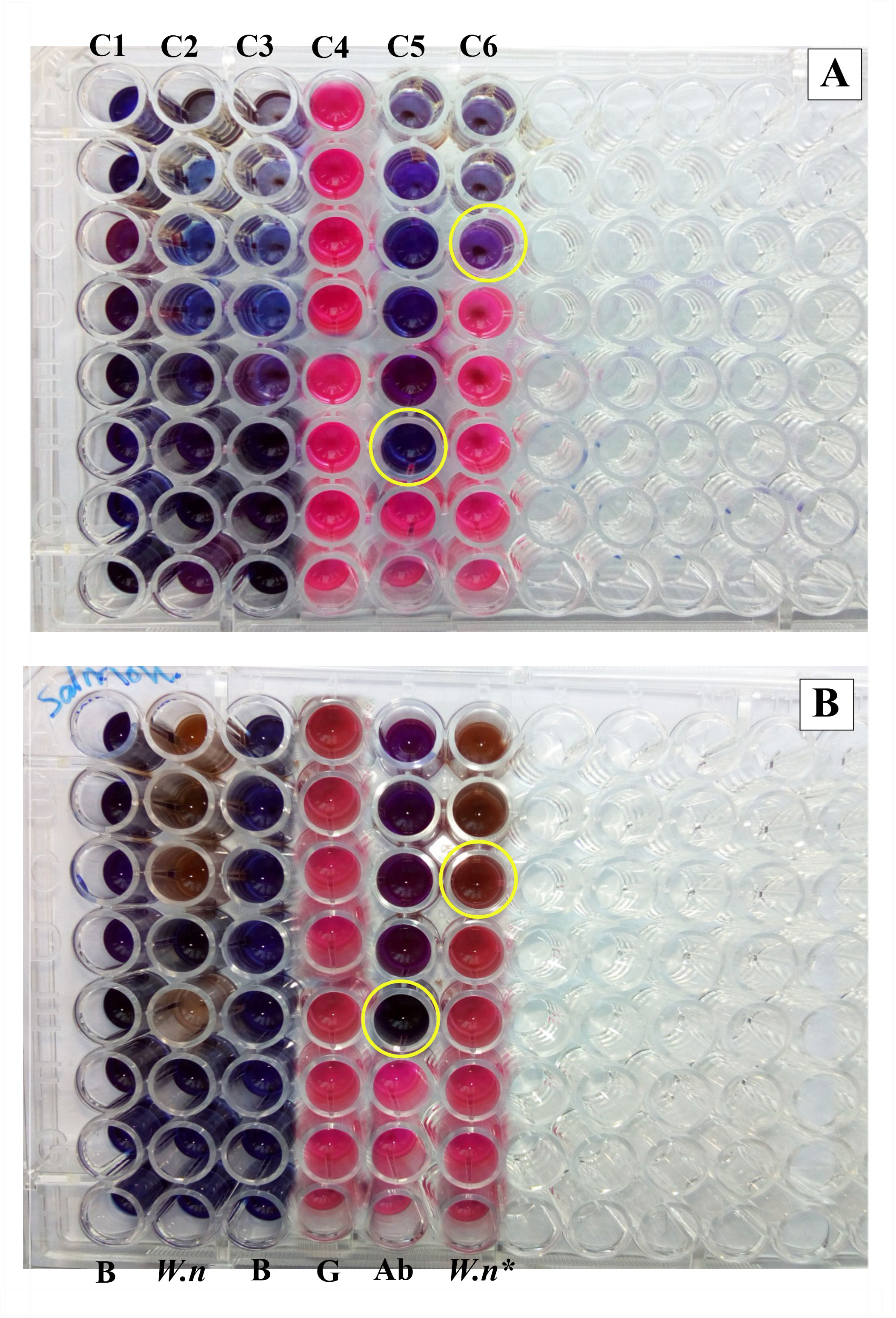

Supplement: Supplementary file 5 — (PNG 5260 kb) [file 42770_2023_1024_Fig6_ESM.png]
